# Tau tubulin kinase 2 is required to initiate mammalian ciliogenesis

**DOI:** 10.1186/2046-2530-1-S1-O18

**Published:** 2012-11-16

**Authors:** S Goetz, KV Anderson

**Affiliations:** 1 Sloan-Kettering Institute, New York, USA

## 

Despite the critical importance of cilia in development and disease, the biochemical pathways that initiate ciliogenesis at the mother centriole are not well understood. Here we describe the identification of a serine-threonine protein kinase, Ttbk2, as an essential component of the ciliary initiation pathway. The mouse mutant *bartleby* (*bby*) was isolated in a genetic screen in our lab based on phenotypes consistent with a severe disruption in Hh signaling, and immunostaining and scanning electron microscopy revealed that *bby* mutants lack cilia. The *bby* phenotype is caused by a nonsense mutation in the gene encoding Tau Tubulin Kinase 2 (*Ttbk2*), truncating the protein within the kinase domain. Independent studies have shown that human mutations in *Ttbk2* are associated with the human neurodegenerative disorder spinocerebellar ataxia type 11. We show that *Ttbk2::GFP* localizes to the distal mother centriole in cells prior to ciliary axoneme extension, and to the transition zone between the basal body and axoneme in ciliated cells. In *Ttbk2bby* mutant cells, components of the IFT complexes, such as IFT88 and IFT140, are not recruited to the transition zone. Thus Ttbk2 acts at a step upstream to IFT in the process of cilia formation and is a critical component of a pathway regulating the initiation of ciliogenesis. Although several kinases have roles in ciliogenesis, Ttbk2 is thus far the only kinase found to have an essential role in cilia formation. Moreover, identification of Ttbk2 substrates and upstream regulators has the potential to reveal other regulators of this important step in ciliogenesis.

